# Community and household socioeconomic factors associated with pesticide-using, small farm household members' health: a multi-level, longitudinal analysis

**DOI:** 10.1186/1475-9276-10-54

**Published:** 2011-11-17

**Authors:** Donald C Cole, Fadya A Orozco, Selahadin Ibrahim, Susitha Wanigaratne

**Affiliations:** 1 Dalla Lana School of Public Health, University of Toronto, Toronto, Ontario, Canada; 2 Instituto de Saúde Coletiva, Universidade Federal da Bahia, Salvador, Brasil & Quito, Ecuador; 3 International Potato Center, Sta Catalina, Pichincha, Ecuador; 4 Institute for Work & Health, Toronto, Ontario, Canada

**Keywords:** cohort, inequalities, developing countries, health promotion, pesticides

## Abstract

**Background:**

Longitudinal studies using multi-level models to examine health inequalities in lower and middle income countries (LMICs) are rare. We explored socio-economic gradients in health among small farm members participating in a pesticide-related health and agriculture program in highland Ecuador.

**Methods:**

We profiled 24 communities through key informant interviews, secondary data (percent of population with unsatisfied basic needs), and intervention implementation indicators. Pre (2005) and post (2007) surveys of the primary household and crop managers included common questions (education, age, and the health outcome - digit span scaled 0-10)) and pesticide-related practice questions specific to each. Household assets and pesticide use variables were shared across managers. We constructed multi-level models predicting 2007 digit span for each manager type, with staged introduction of predictor variables.

**Results:**

376 household managers (79% of 2005 participants) and 380 crop managers (76% of 2005 participants) had complete data for analysis. The most important predictor of 2007 digit span was 2005 digit span: β (Standard Error) of 0.31(0.05) per unit for household and 0.17(0.04) for crop managers. Household asset score was next most important: 0.14(0.06) per unit for household and 0.14(0.05) for crop managers. Community percent with unsatisfied basic needs was associated with reductions in 2007 digit span: -0.04(0.01) per percent for household and -0.03(0.01) for crop managers.

**Conclusions:**

The important roles of life endowments and/or persistent neurotoxicity were exemplified by limited change in the health outcome. Gradients by household assets and community deprivation were indicative of ongoing, structural inequities within this LMIC.

## Background

Over the last decade, equity in health has moved from a preoccupation of concerned researchers [1], human rights activists and some policy makers to a central part of the debate about improving health globally [2]. At the beginning of the decade Starfield defined equity in health as "the absence of systematic [and potentially remediable] differences in one or more aspects of health status across socially, demographically, or geographically defined populations or population subgroups [3]. Others have described the embedded nature of inequities in health and their causes, both proximal and distal [4]. The Commission on Social Determinants of Health contributed to understanding of the generation of inequities and the opportunities for societal responses to them [5].

Country level research in lower and middle income countries (LMICs) has included description, analysis and theoretical developments. The last has been particularly strong in Latin America with a long history of Social Medicine and Collective Health [4,6]. For Ecuador, early description [7] moved through cross-sectional analyses showing strong gradients in child health outcomes [8] and health care utilization [9] to descriptive work on the broad effects of globalization [10] and tracking change in inequalities at the level of parishes [11]. Multi-level modeling has been employed to account for clustering of national survey data [12].

Our own research has focused on smallholder farmers in highland Ecuador. Though we were initially interested in general health, we did not find much variation across farm households. However, neurobehavioral performance did vary substantially across farm households and was associated with farm pesticide use in a measurement intensive cross-sectional survey [13]. Our neurobehavioral measures were part of a World Health Organization battery [14], with proven reliability and validity in measuring neurotoxicity due to organophosphorus and carbamate compounds [15]. These measures are also strongly influenced by lifetime education and age [16]. In a subsequent intervention evaluation, the measures were responsive to reductions in exposure and sub-acute pesticide-related neurotoxicity [17]. In neither of our studies did we find economic gradients in neurobehavioral measures, perhaps due to relative homogeneity within one canton (equivalent to a county). In a subsequent multi-case study across seven communities, we observed systematic variation in child nutrition and pesticide related symptom outcomes by production systems and community social resources [18].

In the Ecosalud II project, upon which this paper is based, analysis of 2005 baseline data from 24 communities in five cantons and three provinces demonstrated variation in pesticide-related knowledge and practices by the proportion of the cantonal population who were literate or indigenous [19]. We also observed community level variation in pesticide use and related health outcomes (poisoning and neurotoxic symptoms) [20]. After interventions designed to reduce pesticide use and improve pesticide-related practices, we used implementation and 2007 follow up survey data from 18 of these communities to examine change in health promotion outcomes by leadership factors across communities and by asset score across households [21]. The extent to which such interventions decreased, left persistent or aggravated inequities across and within communities [22] remained to be examined.

In this paper, we explore the determinants of health inequities in neurobehavioral performance among households in 24 participating communities. Recognizing the difficulties teasing apart inter-related determinants, we nevertheless hypothesized that:

1. Community-level health promotion intervention intensity or coverage and associated pesticide-related practice changes would be associated with better 2007 neurobehavioral performance; and

2. Household assets, community deprivation, and community social resources, would each independently contribute to explaining 2007 neurobehavioral performance.

## Methods

### Design

Cohort of households in 24 communities, analyzed with multi-level, mixed models [23].

### Setting

Selected zones of potato production in highland Ecuador included five cantons or municipalities: Guano, Guamote and Riobamba in the province of Chimborazo (central); Quero in the province of Tungurahua (central); and Montúfar in the province of Carchi (north) (see United Nations approved map at http://www.un.org/Depts/Cartographic/map/profile/ecuador.pdf). Within cantons, communities were identified in conjunction with local agricultural technical assistance partners through a preliminary assessment of potato production volumes. Meetings were held with community leaders regarding interest in agriculture-health interventions and then with the broader population in pre-existing community meetings, as a form of community consent [21].

### Community Level (II) - all independent variables

Profiles were constructed of each participating community through requests for existing information and key informant interviews with community leaders. We obtained the number of households and, as a measure of community social resources, the number of organizations e.g. sport club, parents' group, youth group, irrigation council, farmers' organization, indigenous organization. As these could vary by size of community, we divided them by a denominator of 100's of households to provide a crude community organization indicator (see table 1).

**Table 1:** Community (level II) descriptive statistics (n = 24)

Variables	# of Communities	Continuous (range, median, mean (SD)
Parish proportion of households with unsatisfied basic needs*		
< 0.8	3	
0.8 to < 0.9	11	
0.9+	10	0.59 - 1.0, 0.85, 0.85 (0.13)
Community organizations (#)		
0-1	5	#/100 families
2-3	12	
4-7	7	0 - 7.8, 2.3, 2.9 (2.2)
Community Intervention Scores:		
Coverage^		0-32.7, 20.1, 18.8 (20.1)
Intensity+		0-22, 14.5, 12.8 (8.1)

* Unsatisfied basic needs index is the proportion of the parish population who suffer persistent deficits in at least one of housing (absence of electricity, potable water, sewage, space), health (access to trained health professionals), education (illiteracy, less than primary school), and employment. ^22^

^ Coverage is the average percentage attendance (i.e. per 100 community members) across intervention events.

+ Intensity combines frequency and focus (individual, group or community) of the interventions

To independently characterize communities, we obtained data from the Integrated System of Social Indicators of Ecuador on the unsatisfied basic needs (NBI) indicator, the most comprehensive poverty indicator [24]. Based on data from the 2001 census (the most recent analyzed) and the fifth round of the national living conditions survey, NBI captures the proportion of the population who suffer persistent deficits in at least one of housing (absence of electricity, potable water, sewage, space), health (access to trained health professionals), education (illiteracy, less than primary school), and employment. NBI is the percent with unsatisfied basic needs so higher values indicate more deprivation (see table 1). Unfortunately a parish is the smallest geographical level available and larger than a community (24 communities were within 12 parishes), so the number of communities per parish varied: 1 community in each of 7 parishes; 2 communities in 3 parishes; 3 communities in 1 parish; and 8 communities in an additional parish.

Using Ecosalud II observations during the community interventions, we constructed implementation indices [21]. The intensity index reflected the frequency and focus of the interventions. Frequency was classified as: 1 = once e.g. theatre; 2 = once every 15 days over three months e.g. agricultural workshops; 3 = once every 15 days over 6 months e.g. farmer field school (FFS); and 4 = ongoing e.g. revolving fund. Focus was classified as: 1 = community e.g. field days, revolving funds, health education sessions and theater, 2 = groups where the attendees were members of a farmers' association or were part of concentrated population sector such as schools; and 3 = individual e.g. FFS. The value of the intensity index was the sum of the applicable component values for a particular community. The coverage index was the average of the percentage attendance for each intervention event. The numerator was the average of values extracted from field forms on which health educators and agronomists recorded those present at activities. The denominator was the number of households in the community. Ranges of each index showed good variation across communities (Table 1).

### Level (I): Household and Individual

The baseline survey was carried out from July to September 2005. Approximately twenty volunteer households (range 16-22) were interviewed in each community. Inclusion criteria for participants were: between 18 and 65 years old, literate, lived in the community during the past three years, and interested in participating in the research. In keeping with the Bioethics Committee of the National Health Council of Ecuador standards, participants consented in writing or verbally. Different questionnaires were used for those responsible for managing the household and for those managing crops. To ensure the quality of information, a guide for collecting information was used, data collection was piloted prior to full roll out, and the data collection team supervisor reviewed all surveys for completeness. Supplementary visits were made to clarify or revise incomplete or inaccurate data. A repeat survey was conducted in August 2007, six months after the intervention period (last trimester 2006 to Jan 2007) among the same population of households.

#### Independent variables

For each household, we created a household asset score based on 2005 descriptions of the main materials of the house, roof, and floor; the number of rooms; the number of bedrooms; and the type of land ownership (scored 0 (low, poorer household e.g. with dirt floor, many people per room) to 10 (high, wealthier household with metal roof, more sleeping rooms, owned their own land)). In 2007 we asked whether households had engaged in home improvements since 2005, but few had done so.

At both times, we asked the crop manager about farm pesticide use, measured as the number of applications and active ingredient/application (weight in kilograms per hectare) during the most recent crop cycle for each crop, and summed across crops. Hazardous pesticide type was classified according to the World Health Organization toxicological classification [25] as class Ib (highly hazardous) and class II (moderately hazardous).

Drawing on questions from prior work [26,27] we documented self-reported pesticide-related practices. Household manager practices included: whether clothes used for pesticide spraying were washed with gloves, whether they or other members entered recently sprayed fields, and adequacy of pesticide container disposal. All household manager variables were dichotomized such that better practices (1) were compared to poorer practices (0). Crop manager practices included: washing hands e.g. before eating and before smoking; use of effective personal protective equipment (PPE) for mixing and application e.g. gloves, plastic poncho or rubber pants; and number of body areas usually wet when applying pesticides i.e. more body areas, higher exposure. As the raw ranges of the sum of items for each of these latter indices varied (washing, 4; use of PPE, 5, body areas, 8) we converted each to 0-10 scores. For the first two crop manager scores, higher was better indicating lower pesticide exposure. For the last, higher scores indicated greater pesticide exposure and worse for the person's health.

Age (continuous) and education (dichotomized as less than six and six or more years) were measured in 2005. As alcohol-related problems can affect neurobehavioral performance, we used a 10-item Ecuadorian questionnaire from the Alcoholic Rehabilitation Centre which taps drinking behaviors and social disruption potentially associated with drinking. As positive responses were few, we decided to sum items to provide a composite score for this covariate ranging from 0, no problems, to 10, significant disruption [13].

#### Dependent variable

For our health outcome, we sought a neurobehavioural measure [14] which had been associated with life endowments (education) and neurotoxic pesticide exposure [13] and which could be responsive to health educational interventions aimed at reducing pesticide use and practicing safer pesticide management [17]. Given our scaling up from three communities in our earlier intervention research to twenty four communities in this project, we also needed a measure which was feasible for a lay person to administer reliably and quickly. We therefore chose Digit Span which taps concentration, attention and shorter term memory with good reliability and validity yet which takes a reasonable amount of time [16]. We combined recall of the number of digits, both forwards and backwards, to achieve greater sensitivity, with the sum ranging from 0-no to 12-high recall.

### Analysis

We assessed potential selection biases by comparing characteristics of those lost to follow-up, those participating in both surveys and those excluded due to pairing or missing data (independent t-tests or Chi-square). For categorical practice variables, we constructed a dichotomous improvement variable based on improved practices between surveys. Ordinal scales had sufficient breadth of distributions to be treated as continuous, so difference scores were calculated. The right-skewed distributions of differences in pesticide use required transformation into deciles for modeling purposes, with higher deciles signifying greater reductions in pesticide use. We conducted extensive cross-tabulations of dichotomous and ordinal variables, used ANOVAS and t-tests to link these with continuous variables, and employed correlations among continuous variables to understand patterns in the data.

Multilevel models of predictors of 2007 Digit Span were constructed using the MIXED procedure in SAS Version 9.2 with maximum likelihood estimation [28]. We used the default degrees of freedom method (containment). For covariance we used variance components, i.e. estimation of both the variance of intercept and the variance of the residual, which were uncorrelated. We assessed the variance of the slopes for each predictor variable with an unstructured covariance matrix, but these variances were not significant. We ran separate models for each of the household and crop managers, given different social roles within the household [29] and sets of practice variables. All variables were treated as fixed effects. Starting with an empty model, we first added Digit Span in 2005 (Model I) followed by all individual and household variables (Model II), the latter contribute to testing hypothesis 2. Then we entered the community-level variables (Model III) to test hypotheses 1 & 2. Changes in -2 Log Likelihoods were significant until this point but we were left with a number of variables in which the parameter estimate/standard error ratio was substantially below 1.96 (so not significant at the p < 0.05 level), and a host of non-significant interaction terms which were difficult to interpret. Hence, we removed these non-significant variables in final reduced models (Model IV). Although the change in -2LogLikelihood was not significant, the Akaike Information Criteria (AIC) and Bayesian Information Criteria (BIC) improved at this stage i.e. lower AIC/BIC values, indicating better fit. Though we had less than the suggested minimum sample size of at least 30 groups with 30 observations to detect interactions [23], we did test for both within and cross level interactions in our final models [30]. However, we found no interactions were significant at even a relatively inclusive level of significance (p < 0.1), so we did not include them in the final models.

## Results

### Population

**Figure 1:**
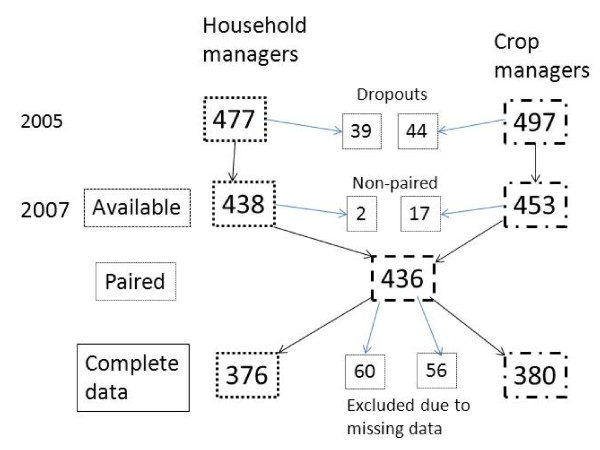
**Analytic cohort selection**.

Among 477 household managers in 2005, 39 (8%) were lost to follow-up (see dropouts in Figure 1), most commonly due to household dissolution, individual migration out of the area, or the project team's persistent inability to locate them. Drop-outs were younger (mean years ± SD of 35 ± 12 versus 40 ± 12 in cohort, unpaired t-test p = 0.03), and had better digit span scores (4.6 ± 1.8 versus 4.1 ± 1.7, p = 0.07) than cohort participants. Household managers were overwhelmingly women (427/438, 97%). Among 497 crop managers in 2005, 44 (9%) were lost for similar reasons. Drop-outs were also younger (37 ± 13 versus 42 ± 13, p = 0.02) and had a lower proportion with worse alcohol-related problems score (0.16 vs. 0.4, p = 0.002). Crop managers were primarily men (396/453, 87%).

In order to use asset information (on the household manager questionnaire) for crop managers and pesticide use information (on the crop manager questionnaire) for household managers, we excluded 2007 households in which only one was present. We paired the household and crop managers for each household (n = 436 households) (see paired in Figure 1). Missings created challenges for several variables, citing the largest numbers here: age, 40 household and 14 crop managers; education, 37 household and 48 crop managers; 2005 digit span, 36 household and 49 crop managers; practice variables, 31 household and 18 crop managers. We were left with 376 household managers (79% of 2005, 86% of available 2007 participants) and 380 crop managers (76% of 2005, 85% of available 2007 participants) with complete data. Differences between those excluded for multiple missing values and those retained were only significant for: higher mean 2005 digit span (4.5 versus 4.1, p = 0.02) and lower mean reduction in highly hazardous pesticide use (-0.5 versus -1.0, p = 0.02) among excluded household managers; and lower mean alcohol-related problems score (1.1 versus 1.5, p = 0.001) among excluded crop managers.

### Practices

On univariate analyses including all those with responses for that variable, several reported practices changed. A significant improvement was noted in the proportion of household managers reporting wearing gloves to wash spray clothing (0.32 to 0.55, McNemar p < 0.0001). Among crop managers, highly significant improvements (all paired t-tests p < 0.0001) were found in the use of PPE (mean ± SD score of 3.9 ± 1.9 to 4.4 ± 1.8), reduced wetting of the body (5.2 ± 2.3 to 3.9 ± 2.3) and washing face and hands (8.1 ± 1.9 to 9 ± 3.3). Use of highly hazardous (2.0 ± 4.3 to 0.7 ± 1.8 kg/hectare/crop cycle) and moderately hazardous (1.7 ± 3.8 to 0.7 ± 1.8) pesticides also decreased on cohort farms. Among those managers with complete data, overall patterns of change remained the same (see change scores in Table 2)

**Table 2:** Household and individual (both level I) descriptive statistics by domain and social role

Domain and Variables	Household manager (n = 376)	Crop Manager (n = 380)
Household Asset Score (2005) (0-10; mean (SD))	7.2 (1.4)	7.2 (1.4)
Household pesticide use (kg/hectare)*(2007 minus 2005; mean, (SD)):		
Highly hazardous pesticide use	-1.5 (4.6)	-1.3 (4.6)
Moderately hazardous pesticide use	-1.1 (4.2)	-1.0 (4.1)
Pesticide-related Practice Changes		
Proportion improving practice (2005 to 2007):		
- Washing application clothes with gloves	212 (0.56)	Not applicable
- Entering recently sprayed fields	149 (0.40)	Not applicable
- Disposing of pesticide containers	255 (0.68)	Not applicable
Difference scores (2007 minus 2005; mean, (SD)):		
- Washing hands & face (0 low-10 high)	Not applicable	0.93 (3.8)
- PPE use (0 none-10 all)	Not applicable	0.60 (2.3)
- Wetting body areas (0 none-10 all)	Not applicable	-1.1 (2.7)
Lifecourse and health predictors (all 2005)		
Age (yrs, mean SD)	39.8 (12.3)	41.8 (13.1)
Education < 6 years (n,%)	124 (33.0%)	103 (27.1%)
Alcohol-related problems score (0-10, mean (SD))	1.1 (0.28)	1.45 (0.5)
Digit Span (neurobehavioural performance indicator)		
2005 (0-12, mean (SD))	4.1 (1.6)	4.4 (1.6)
2007 (0-12, mean (SD))	4.2 (1.7)	4.4 (1.4)

*Although use of pesticides is a household variable, it is assigned to each manager and their n's are different, so values vary across columns

### Models

In each set of managers' models (see tables 3 &4), digit span in 2005 played a substantial role in explaining variation in 2007, signaled by both the change in -2LogLikelihood and the coefficients e.g. 0.38 increase in 2007 with each 1.0 increment in 2005 for the household manager. The additional effects of level I variables were also important, particularly age (significant reductions per year).

**Table 3:** Mixed multiple regression models of digit span for Household manager (n = 376)

	Intercept only β (SE)	Model I Level 1- baseline β (SE)	Model II Level I- other β (SE)	Model III Level II β (SE)	Model IV Reduced β (SE)
**Level I (individual/household)**					
Intercept	4.14 (.19)*	2.61 (.25)*	3.40 (.60)*	6.21 (1.0)*	6.90 (.96)*
Digit Span in 2005		.38 (.05)*	.30 (.05)*	.29 (.05)*	.28 (.05)*
					
Age (yrs)			-.04 (.01)*	-.04 (.01)*	-.03 (.01)*
Education < 6 years (yes vs. no)			.19 (.17)	.17 (.17)	
Alcohol-related problems score			.19 (.26)	.21 (.26)	
Household Asset Score (2005)			.12 (.06)*	.16 (.06)*	.13 (.06)*
					
**Practice Changes (05 to 07)**					
Washing application clothes with gloves improvement			-.14 (.15)	-.14 (.15)	
Entering sprayed fields improvement			.04 (.15)	.02 (.15)	
Disposing of pesticide containers more adequate			-.15 (.15)	-.14 (.15)	
Highly hazardous pesticide use reduction (per decile)			.01 (.02)	.02 (.02)	.03 (.015)*
Moderately hazardous pesticide use reduction (per decile)			.04 (.02)	.04 (.02)	
					
**Level II (parish/community)**					
Proportion households with unsatisfied basic necessities				-.03 (.01)*	-.04(.01)*
Community organizations /100 families				.06 (.06)	
Intervention intensity				-.02 (.03)	
Intervention coverage				-.01 (.02)	
					
**Covariance Parameter Estimates**					
intercept	.70 (.24)	.45 (.17)	.48 (.18)	.17 (.09)	.22 (.10)
residual	2.21 (.17)	1.92 (.15)	1.72 (.13)	1.72 (.13)	1.76 (.13)
					
**Model comparison^**					
-2Log Likelihood	1408.3	1349.9	1311.7	1294.5	1306.0
Change in -2LogL (df)		-58.4 (1)	-38.2 (9)	-17.2(4)	11.5 (9)
P-value for change in -2LogL		< .0001	< .0001	.0018	.2430
AIC	1414.3	1357.9	1337.7	1328.5	1322.0
BIC	1417.8	1362.6	1353.0	1348.6	1331.5

Bolded values* significant at p < 0.05

^AIC = Akaike Information Criteria, BIC = Bayesian Information Criteria, both measures of fit

**Table 4:** Mixed multiple regression models of digit span for Crop manager (n = 380)

	Intercept only β (SE)	Model I Level 1- baseline β (SE)	Model II Level I- other β (SE)	Model III Level II β (SE)	Model IV Reduced β (SE)
**Level I (individual/household)**					
Intercept	4.42 (.13)*	3.40 (.23)*	3.90 (.50)*	6.26 (.76)*	6.40 (.77)*
Digit Span in 2005		.23 (.04)*	.17 (.04)*	.16 (.04)*	.17 (.04)*
					
Age (yrs)			-.03 (.01)*	-.03 (.01)*	-.03 (.01)*
Education < 6 years (yes vs. no)			-.22 (.17)	-.27 (.17)	
Alcohol-related problems score			.03 (.14)	.06 (.13)	
Household Asset Score (2005)			.11 (.05)*	.15 (.05)*	.14 (.05)*
					
**Practice Changes (05 to 07)**					
Washing hands & face improvement			.05 (.02)*	.04 (.02)*	.04 (.02)*
PPE use improvement			-.06 (.03)*	-.06 (.03)*	-.07 (.03)*
Wetting body areas reduction			.03 (.03)	.03 (.03)	
Highly hazardous pesticide use reduction (per decile)			-.01 (.02)	-.01 (.02)	
Moderately hazardous pesticide use reduction (per decile)			-.02 (.02)	-.01 (.02)	
					
**Level II (parish/community)**					
Proportion of households with unsatisfied basic needs				-.03 (.01)*	-.03 (.01)*
Community organizations/100 families				-.02 (.04)	
Intervention intensity				-.01 (.02)	
Intervention coverage				.02 (.01)*	.02 (.01)*
					
**Covariance Parameter Estimates**					
intercept	.27 (.11)	.25 (.10)	.19 (.08)	.06 (.05)	.08 (.05)
residual	1.80 (.13)	1.68 (.13)	1.5 (.11)	1.53 (.12)	1.54 (.12)
					
**Model comparison^**					
-2Log Likelihood	1330.8	1304.6	1263.5	1250.4	1256.2
Change in -2LogL (df)		-26.2(1)	-41.1(9)	-13.1(4)	5.8 (7)
P-value for change in -2LogL		< .0001	< .0001	.0108	.5632
AIC	1336.8	1312.6	1289.5	1284.4	1276.8
BIC	1340.3	1317.3	1304.8	1304.4	1288.0

Practice changes had small influences on digit span: reduction in moderately hazardous pesticide use of one decile was associated with an increase in digit span of 0.04 among household managers, while improved washing of the hands and face after application by crop managers was of similar magnitude. Improvement in PPE use, negatively associated with digit span among crop managers, may be due to poor quality, infrequent cleaning, inappropriate use or lack of filter changes in PPE. Community coverage by health promotion interventions was associated with a modest increment in digit span (0.2) only among the crop managers.

Both household assets (increases) and the community proportion of households with unsatisfied basic needs (decreases) were significant contributors to 2007 digit span. The latter effect was primarily additional, as very few level 1 coefficients were reduced, many stayed the same and some even increased, such as household asset score.

## Discussion

Each of our hypotheses was proven at least partially correct, with some differences between manager roles and some limits to explanation possible. The limited change in mean digit span between survey times likely indicates the influence of broader determinants of health, the stability of the measure, the persistence of pesticide-related neurotoxic effects, and/or limited intervention impacts. For the first, lower digit span was associated with lower education but also increasing age [14], the latter a reflection of lifetime conditions or declining ability. For the second, cross-survey correlations were of medium strength, so additional variance could be accounted for by repeat measurement. Unfortunately, the limited number of neurobehavioural measures applicable in low education populations and limited resources impeded our examining this among a larger set of neurobehavioral measures. For the third, most participants had worked in agriculture since childhood, with several decades of pesticide exposure. Cumulative years as well as recent experience working with pesticides are both thought to contribute to neurobehavioral deficits [31]. Unlike long duration cohort studies showing Parkinson's Disease and Alzheimer's disease impacts of such exposure in the elderly [32], shorter duration cohort studies of neurobehavioral performance are rare.

Regarding the fourth explanation (and our first hypothesis), only a few intervention evaluations exist, including our earlier work in which more intensive interventions resulted in more substantial improvements [17]. Community intervention coverage was important for crop managers, being more involved in agricultural interventions, while household managers had difficulties participating in activities [21,29]. Hence hypothesis 1 was only partially supported, due to the multifactorial determination of neurobehavioral performance, the chronic nature of neurotoxic effects, and the limited intensity of our interventions.

For hypothesis 2, household assets (positive) and community prevalence of unsatisfied basic needs (negative) both explained variation in 2007 neurobehavioral performance. Rural areas of Ecuador have not experienced the increases in economic consumption occurring in urban areas [11]. Substantial differences in social capital and access to resources occur within as well as across geographic areas [33]. Heterogeneity in social networks and agricultural production methods has been demonstrated in Carchi [34]. From the mid-90's to the mid 2000's, gradients in resources were either persistent (Carchi parishes) or worsened (Chimborazo province), particularly in indigenous cantons (Guamote, Riobamba) (p 26) [11]. We can describe these heterogeneities as inequities [1] attributable to the combined effects of: a historical legacy of hierarchy and social stratification [4]; persistent ignoring of farmers' rights by politicians and farmers [19]; in-egalitarian social policy for decades [35]; declining productivity among smallholder farmers [36]; and unequal globalization [10], a widespread underlying reason for inequities in health [37]. Our study demonstrates inequities at a more micro level to complement others' more macro findings [7-9,11].

On a cautionary note, the need for pairing and missing data reduced our cohort with full data considerably, despite adequate follow-up. Our use of self-report and recall, rather than direct observation and bi-weekly logs [26,27], likely reduced pesticide use data validity. Respondents aware of the broader sustainable and health agriculture goals of EcoSalud II may have reported greater changes than actually occurred. Our community social resources measure, only the number of organizations, does not reflect broader organization types or contributions. We focused on literate populations, in order to use our neurobehavioural measure, and worked with community leadership who were open to cooperating with the project and rural small-holder farm members who agreed to participate in interventions. Hence our communities and households are necessarily selected samples, with only part of the full spectrum of inequities which exist in rural Ecuador or other LMICs. Our results on community deprivation and household assets may be less dramatic than one might expect to exist among those covering a broader range of the spectrum of inequity.

## Conclusions

Further work should follow our cohort through the period of social policy development occurring in Ecuador, including restrictions on the use of highly hazardous pesticides (July 2010) and implementation of a economic supports for the poor. Combined primary and secondary research in LMICs should continue exploring community or neighborhood level social gradients and their consequences for different health outcomes [38] and interventions [39]. Exploration of the role of rural municipalities in addressing inequities in determinants of health [40] including monitoring of policy adherence [19] should be encouraged. Finally, correcting the current imbalance in social inequities research between High and Low-Medium Income Countries is urgently needed to inform global and national social policies addressing inequities [5].

## Abbreviations

AIC: Akaike Information Criteria; BIC: Bayesian Information Criteria; LMICs: lower and middle income countries; NBI: 'necessidades basicas insatisfechas' or unsatisfied basic needs indicator; PPE: personal protective equipment.

## Competing interests

The authors declare that they have no competing interests.

## Authors' contributions

FO co-designed the research, oversaw all data collection, participated in analysis and interpretation, wrote sections, and iteratively revised the manuscript. DCC co-designed the research, participated in analysis and interpretation, wrote the initial draft, and iteratively revised the manuscript. SI co-led the analysis, assisted with interpretation, and revisions. SW co-led the analysis, assisted with interpretation, writing and revisions. All authors read and approved the final manuscript.
